# Urinary Polyamines: A Pilot Study on Their Roles as Prostate Cancer Detection Biomarkers

**DOI:** 10.1371/journal.pone.0162217

**Published:** 2016-09-06

**Authors:** Tik-Hung Tsoi, Chi-Fai Chan, Wai-Lun Chan, Ka-Fung Chiu, Wing-Tak Wong, Chi-Fai Ng, Ka-Leung Wong

**Affiliations:** 1 Department of Applied Biology and Chemical Technology, The Hong Kong Polytechnic University, Hung Hom, Hong Kong SAR; 2 Department of Chemistry, National University of Singapore, 21 Lower Kent Ridge Road, Singapore, Singapore; 3 SH Ho Urology Centre, Division of Urology, Department of Surgery, The Chinese University of Hong Kong, Shatin, N.T., Hong Kong SAR; 4 Department of Chemistry, Hong Kong Baptist University, Kowloon Tong, Hong Kong SAR; ENEA Centro Ricerche Casaccia, ITALY

## Abstract

Current screening methods towards prostate cancer (PCa) are not without limitations. Research work has been on-going to assess if there are other better tests suitable for primary or secondary screening of PCa to supplement the serum prostate specific antigen (PSA) test, which fails to work accurately in a grey zone of 4-10ng/ml. In this pilot study, the potential roles of urinary polyamines as prostate cancer biomarkers were evaluated. PCa, benign prostatic hyperplasia (BPH) patients and healthy controls (HC) showing PSA>4.0ng/ml were enrolled in the study. Their urine samples were obtained, and the urinary levels of putrescine (Put), spermidine (Spd) and spermine (Spm) were determined by ultra-high performance liquid chromatography coupled with triple quadrupole mass spectrometer (UPLC-MS/MS). Receiver operating characteristics (ROC) curve and Student’s t-test were used to evaluate their diagnostic accuracies. Among the three biogenic polyamines, Spm had demonstrated a good diagnostic performance when comparing their levels in PCa patients with BPH patients (1.47 in PCa vs 5.87 in BPH; *p*<0.0001). Results are in accordance with transrectal ultrasound prostatic biopsy (TRUSPB) results, with an area under curve (AUC) value of 0.83±0.03. Therefore urinary Spm shows potential to serve as a novel PCa diagnostic biomarker, which in turn can help to address the limited sensitivity and specificity problem of serum PSA test.

## Introduction

PCa is one of the most common non-skin male-related cancers in the world, and it is one of the leading causes of mortality and momentous public health impact in many developed countries, like most western European nations and the United States [1–2]. No exception is Hong Kong in this public health issue. With reference to the statistics of Hong Kong Cancer Registry, Hospital Authority, HKSAR, PCa is ranked 3^rd^ in the most common cancers in men and 5^th^ in the most fatal cancers. Given the latency of early, treatable PCa and the lethality in its late and discernible stage, there is an urgent demand for more sensitive and accurate diagnostic methods to detect early PCa so that the treatment outcome can be significantly improved and more lives being saved.

Current diagnosis of PCa relied mainly on clinical suspiciousness raised by digital rectal examination (DRE) and serum PSA test, followed by TRUSPB confirmation. Although DRE is a simple procedure, it brings discomfort to patients. It is also a strongly-investigator-dependent technique, showing poor accuracy for PCa diagnosis. [3] In particular, DRE is not a good tool for the early detection of PCa because most DRE positive PCa results are of advanced staging. While PSA test is also a simple and popular test with good sensitivity to detect early cancer; however, elevated PSA levels had also been observed in patients with BPH and prostatitis, etc., which means it has a poor specificity towards PCa. For reference, within the grey zone of 4-10ng/ml, the positive-predictive value has a small mean value of 21%. [4] A wide variety of PSA concepts, like the PSA density of transition zone [5–6], free/total PSA ratio [6–7], p2PSA and Prostate Health Index, [8–9] were introduced and tried to improve the performance of PSA measurement. TRUSPB is currently the main diagnostic approach for histological confirmation of prostate cancer. However, its procedures are tedious and lead to significant discomfort and complications to patients. [10–12] As a result of the poor specificity of serum PSA test, many patients without PCa are subjected to TRUSPB and thus its potential complications. It is therefore essential to develop more efficient detection kits for accurate PCa screening at the earlier stages for the sake of well-being.

Urine-based assays present an important area for biomarker research. Urine collection is non-invasive and readily available; more importantly, it affords detection samples for either malignancy-related metabolites excreted in urine, directly exfoliated cancer cells or prostatic products secreted into the genitourinary tract. [13] This is why it is viewed as a perfect source for prostate cancer biomarkers investigation. Currently there have been many urinary PCa specific biomarkers developed under clinical review. [13–15]

One example of such cancer biomarkers are natural polyamines. Interests on these analytes have been starting in 1971 when Russell reported a considerable increase of urinary polyamines such as Put, Spd and Spm in patients with various types of solid tumors and leukaemias. [16] Afterwards, polyamine studies focusing on specific cancers continued, like cervical cancer [17], colorectal cancer [18] and breast cancer [19], etc. Nevertheless, very scanty articles discussed the relationship between urinary polyamines levels with prostate cancer. The prostate indeed is one of the human tissues having the highest concentrations of polyamines, especially Spm. [20] Several studies on polyamines and PCa had shown the clinical relevance of polyamines in prostatic cancer cells’ proliferation and differentiation under the control of androgens, and interference with their homeostasis may serve as a target for PCa chemotherapy. [20] Some studies also suggested the role of prostatic Spm concentration as a biomarker for diagnosing PCa owing to the observation of its significantly different levels in non-malignant and malignant tissues. [21] All of these researches supported the idea of monitoring urinary polyamines concentration to be a means for PCa diagnosis.

In this pilot study, we attempted to evaluate the potential of three main urinary polyamines (Put, Spd and Spm) as biomarkers for PCa detection by comparing the cases between diagnosed PCa, BPH patients and HC. Through a well validated chromatographic method, urinary Spm had been shown to possess usefulness in differentiating PCa from non-cancerous cases including BPH, and it could help to act as a secondary screening tool to serum PSA test to address its high false-positive rate when using 4.0ng/ml as a cut-off point.

## Materials and Methods

### Clinical samples

This study was reviewed and approved by the Clinical Research Ethical Committee of the Chinese University of Hong Kong, and it was performed strictly according to the guidelines developed by that committee. Written consent was acquired from all of the patients. Urine samples were obtained at noon time after lunch prior to prostatic biopsy from 165 male patients (age > 50) having serum PSA level greater than 4.0ng/ml between Oct-2014 and Mar-2016. These patients’ urine samples were accepted only when they didn’t have clinically active urinary tract infection which might pose a biasing effect. When patients did not consent to take part in the study, or they showed clinical evidence of other types of cancers, they were excluded from our sampling scheme.

Amongst these 165 patients, there were three subsets for the clinical samples enrolled in the current study. 66 were diagnosed to have PCa and the remaining 99 were evaluated as having no evidence of malignancy (NEM) by using TRUSPB as the reference standard. To further categorize these 99 NEM patients, using the criteria of prostate volume >30ml, 88 were found to have BPH while others were considered as HC. All pathological examinations were conducted at Prince of Wales Hospital, The Chinese University of Hong Kong, Hong Kong under supervision of experienced uro-pathologists. Table 1 shows all the clinicopathologic characteristics of samples. All samples were stored at -20°C until measurement. All measurements were conducted within three months after collection.

**Table 1:** Clinicopathologic characteristics of patients.

Characteristics	PCa (n = 66)	BPH (n = 88)	HC (n = 11)	*p* value (PCa vs BPH)	*p* value (PCa vs HC)	*p* value (BPH vs HC)
**Age (years)**						
Mean (SEM)	69.6 (0.8)	66.9 (0.6)	64.9 (1.1)	0.018	0.027	0.245
Median	69	66	65			
Range	54–86	51–79	59–74			
**Preoperative PSA (ng/ml)**						
Mean (SEM)	46.39 (8.61)	12.39 (1.57)	26.54 (7.51)	<0.0001	0.350	0.007
Median	15.60	8.60	8.50			
Range	4.20–299.00	4.40–98.50	4.30–66.00			
**Gleason score (GS)**						
5	1					
6	26					
7	15					
8	10					
9	12					
10	2					
**Prostate volume (ml)**						
Mean (SEM)	43.81 (2.44)	67.28 (2.98)	17.46 (2.67)	<0.0001	<0.0001	<0.0001
Median	40.00	56.50	20.40			
Range	16.60–87.80	32.20–162.00	4.60–30.00			

SEM represents the standard error of the mean.

### Materials and chemicals

Methanol was obtained from TEDIA (HPLC/Spectro grade, ≥ 99.9%). Acetonitrile was obtained from ACS (HPLC grade, ≥ 99.9%). Water was purified in a MilliQ Direct Water Purification System (Millipore, USA). All standard compounds, including 1,4-Diaminobutane (Put, 99%), spermidine (Spd, ≥99.0%), spermine (Spm, ≥99.0%), 1,4-Diamino(butane-d_8_) dihydrochloride (98 atom % D), spermidine–(butane-d_8_) trihydrochloride (98 atom % D, 95% CP), spermine–(butane-d_8_) tetrahydrochloride (97 atom % D, 95% CP) and heptafluorobutyric acid (HFBA, ≥99.0%) were purchased from Sigma-Aldrich (Hong Kong, China) and used without further purification. Strong Anion Exchange solid phase extraction (SPE) cartridges were obtained from Phenomenex (Strata, 100mg/3mL, USA). Centrifugation was performed using a Refrigerated centrifuge obtained from Eppendorf (5417R, Hong Kong, China).

### Determination of creatinine

The creatinine concentration inside urine samples were determined by LabAssay^™^ Creatinine assay (Wako, Japan). Briefly, urine samples and standards were thawed, deproteinized and centrifuged. The supernatant was separated and reacted with picric acid in alkaline solution to produce tangerine condensate through Jaffe reaction. [22] Quantitation of total creatinine inside samples was made by measurement of absorbance by a Clariostar Monochromator Microplate Reader (BMG Labtech, Hong Kong). Concentrated urine samples which exceeded the calibration points were diluted with water with appropriate dilution factors before sample preparation. Each sample was determined at least twice with RSD less than 15%.

### Standard preparation for determination of polyamines

Stock solutions (5000μg/ml) of each polyamine (Put, Spm, Spd) were prepared in water separately. The three stock solutions were mixed and diluted to give an intermediate standard (50μg/ml), which was then used to prepare a series of working standards with polyamine concentrations of 10, 25, 50, 100, 250, 500, 1000ng/ml in water. For internal standards, the stock solutions (5000μg/ml) of each polyamine (Put-d_8_, Spm-d_8_, Spd-d_8_) were prepared in water individually. The three stock solutions were mixed and diluted to give an IS working solution (1μg/ml) in water.

### Sample/standard pretreatment for determination of polyamines

The sample preparation procedures followed the method developed by Häkkinen *et al*. with little modifications. [23] Firstly urine samples/standards were thawed naturally and centrifuged for 5 min at 13000 rpm and room temperature. 120 μL of urine sample/standard supernatant and 60 μL of IS working solution were mixed with 420 μL of water. 550 μL of this well-mixed solution was passed through the SPE cartridges, which had been conditioned and equilibrated with 1mL of methanol and water respectively. 450 μL of water was passed through the cartridge afterwards to elute out all polyamines. 400 μL of these SPE treated samples were then mixed with 100 μL of 10% HFBA, and the final mixture was ready for instrumental analysis. Concentrated urine samples which exceeded the calibration points were diluted with water with appropriate dilution factors before sample preparation.

### Quality control samples for determination of polyamines

For each batch of sample analysis, three Quality control (QC) working solutions were analyzed to verify the accuracy of calibration curves and ensure comparability among batches. The solutions were prepared using analyzed control urine samples from our research group. The polyamines concentrations of controls’ urine samples were determined and then mixed equally to give a pooled urine sample. Afterwards, three QC working solutions with different polyamine concentration ranges (low, medium and high) were prepared by mixing this pooled urine sample with standard solutions. For low QC working solution, the SPE-treated pooled urine sample was mixed with SPE-treated 10ng/ml standard in a 1:7 ratio. For medium QC working solution, the SPE-treated pooled urine samples were mixed with SPE-treated 100 ng/ml standard in a 1:1 ratio. For high QC working solution, the SPE-treated pooled urine sample was mixed with SPE-treated 1000ng/ml standard in a 1:1 ratio.

### Stability studies

For stability studies, Häkkinen *et al*. had previously demonstrated that both the standard mixtures and QC samples were stable after storing at six hours at room temperature (short-term stability), after storage at -20°C and -80°C for two months (long-term stability) and after going three cycles of freezing and thawing before sample preparation (freeze thaw stability). [23] For further verification, we have tried to analyze both the content of polyamines and creatinine inside both standards and selected urine samples. It was found that, upon five cycles of freeze and thaw, all the contents were still stable in six months’ time when stored at -20°C. For the SPE-treated samples, it was stable for at least two days when stored at 4°C and up to a year when stored at -20°C.

### Instrumentation and statistical analysis

The quantitation of polyamines was performed by Ultra-high Performance Liquid Chromatography coupled with a triple quadrupole mass spectrometer (UPLC-MS/MS). LC separation was done by an Agilent 1290 Infinity Quaternary LC System while mass analysis was done by an Agilent 6460 Triple Quadrupole mass spectrometer equipped with an Agilent Jet Stream technology electrospray ionization source. The column used was an Agilent EclipsePlus C18 RRHD (2.1x50 mm, 1.8 μm) protected with an Agilent SB-C18 guard column (2.1x5 mm, 1.8 μm).

The LC elution profiles were optimized as follows: Eluent A was water with 0.1% HFBA while eluent B was acetonitrile with 0.1% HFBA. Eluent A was decreased from 95% to 60% in 10 minutes, and from 60% to 10% in 1 minute. Afterwards the gradient was held constant for 5 minutes. The gradient was then increased from 10% to 95% in 1 minute, and held constant for 8 additional minutes. (Total run-time = 25 minutes)

The autosampler and column temperatures were set at 4 and 35°C respectively. Injection was achieved by 5-second needle wash in Flush Port mode for 3 times with eluent B. 10 μL was injected each time.

For the source parameter, drying gas (nitrogen) temperature was set as 300°C with 5 L/min flow rate. Nebulizer pressure was 45 psi. Sheath gas temperature was set as 250°C with 11 L/min flow rate. Capillary voltage was set as 3500V. For mass detection, scheduled multiple reaction monitoring (MRM) was performed. The information of MRM transitions can be found in S1 Table.

The result was calculated using Agilent MassHunter Workstation software. Calibration curves were fitted linearly without any weighing. The correlation coefficients should not be smaller than 0.995. Acceptance values for each calibration points and quality control working solutions were ±30% to ensure accuracy. For precision verification, after every 10-sample injection, a 250ng/ml standard was injected and checked if it can be reproduced (±15%).

For statistical analysis, the ROC curve and the AUC were obtained by using GraphPad Prism 6 (GraphPad Software, San Diego, CA, USA). A *p* value smaller than 0.05 (two-tailed) was considered as statistically significant during comparison based on Student’s t-test.

## Results

### Urinary polyamines content

**Fig 1:**
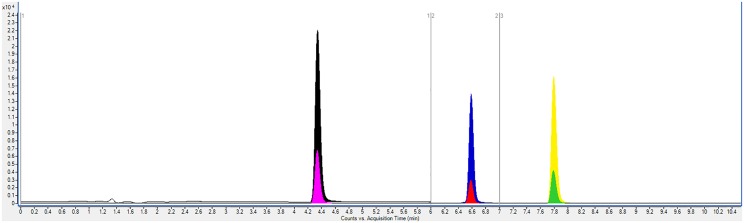
Overlaid UPLC-MS/MS SRM chromatograms of 1000 ppb mixed polyamines standard (0–10 mins being shown). Put (Black peak, t_R_ = 4.3 min), Put-d_8_ (Pink peak, t_R_ = 4.3 min), Spd (Blue peak, t_R_ = 6.6 min), Spd-d_8_ (Red peak, t_R_ = 6.6 min), Spm (Yellow peak, t_R_ = 7.9 min) and Spm-d_8_ (Green peak, t_R_ = 7.9 min).

Put, Spd, Spm and their corresponding deuterated internal standards were successfully separated and quantified from all samples by UPLC-MS/MS. (Fig 1) The calibration curves were all satisfactory with r^2^ not less than 0.995 (See S1 Fig), and all QC measures were passed, guaranteeing comparability between samples analyzed on different days. The mean urinary polyamines concentration for each patient was then normalized to their urinary creatinine levels and expressed as μmol/g of creatinine. (See S2 Table for creatinine results) This is to compensate for any diuresis processes hindering actual quantity measurements. [24]

**Fig 2:**
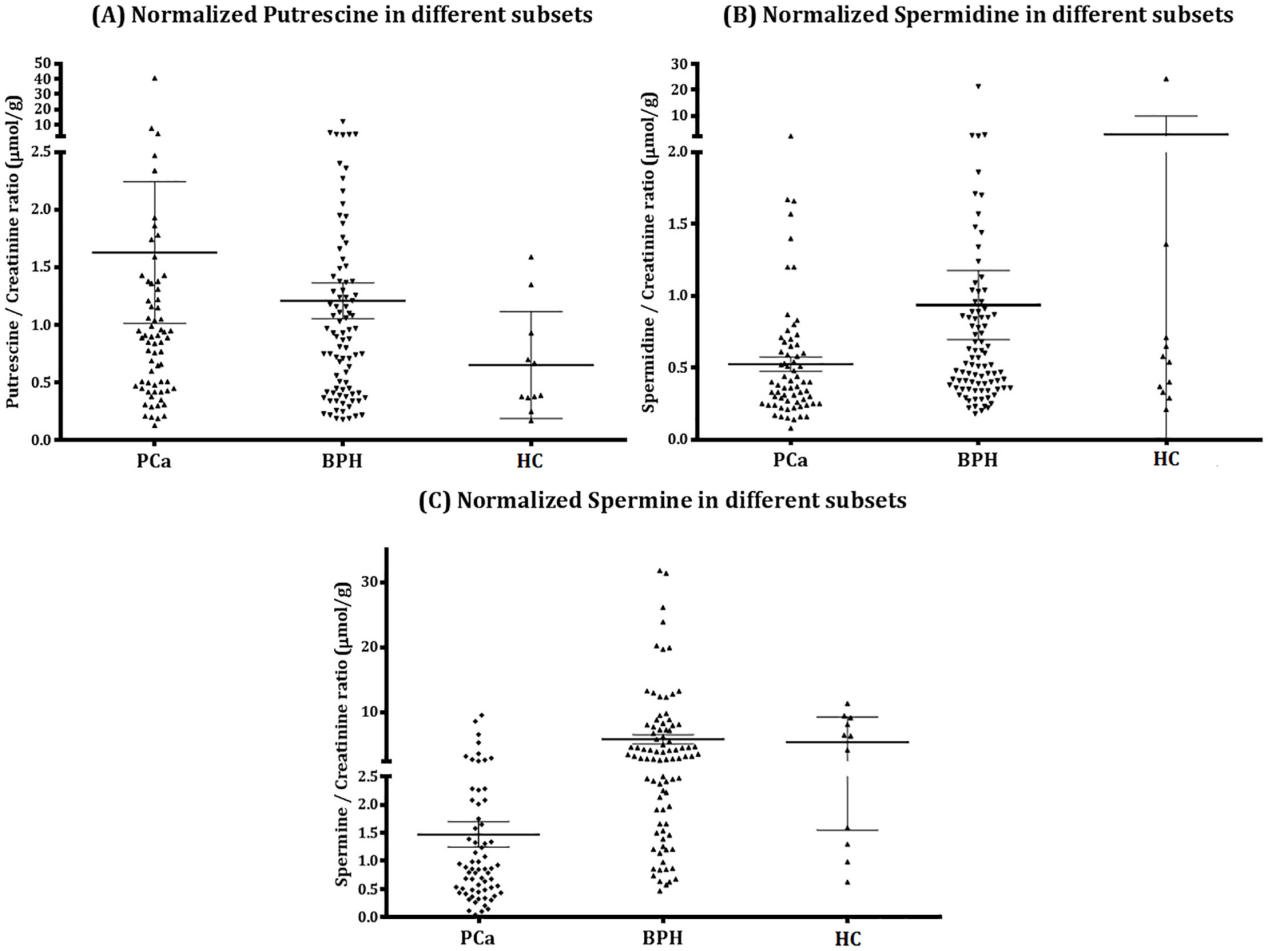
Distribution of (A) normalized Put, (B) normalized Spd, (C) normalized Spm values in PCa, BPH and HC. The black bar in the figures indicates the mean value of each subset while the error bar indicates the corresponding SEM.

Table 2 and Fig 2 showed the data and graphical comparison of different subsets’ normalized polyamines levels.

**Table 2:** Column statistics of normalized polyamine contents (μmol/g of creatinine) in different subsets.

Polyamine contents	PCa (n = 66)	BPH (n = 88)	HC (n = 11)	*p* value (PCa vs BPH)	*p* value (PCa vs HC)	*p* value (BPH vs HC)
**Normalized Put**						
Mean (SEM)	1.63 (0.61)	1.21 (0.16)	0.65 (0.14)	0.459	0.522	0.212
Median	0.87	0.92	0.39			
Range	0.13–40.64	0.18–12.04	0.17–1.59			
25% Percentile	0.45	0.41	0.37			
75% Percentile	1.24	1.38	0.93			
**Normalized Spd**						
Mean (SEM)	0.52 (0.05)	0.94 (0.24)	2.71 (2.17)	0.147	0.014	0.081
Median	0.39	0.52	0.54			
Range	0.08–2.09	0.18–21.42	0.21–24.40			
25% Percentile	0.25	0.36	0.33			
75% Percentile	0.65	0.89	0.71			
**Normalized Spm**						
Mean (SEM)	1.47 (0.22)	5.87 (0.71)	5.43 (1.17)	<0.0001	<0.0001	0.833
Median	0.86	3.25	6.37			
Range	0.05–9.57	0.47–31.78	0.63–11.36			
25% Percentile	0.48	1.72	1.30			
75% Percentile	1.82	7.65	9.18			

SEM represents the standard error of the mean.

Among the three polyamines monitored, normalized Spm showed a significant decrease in PCa patients compared to non-cancerous cases including BPH patients and HC in terms of statistical means (Unpaired student’s t-test). In detail, the mean value was 1.47 in PCa vs 5.87 in BPH vs 5.43 in HC. *p* values were <0.0001 in t-test, which means significant differences at the pre-set criteria of *p* <0.05. For normalized Put and Spd, no obvious enhancement or suppression could be observed neither by looking at their distributions or comparing their mean values by t-test. (Put: 1.63 in PCa vs 1.21 in BPH vs 0.65 in HC; Spd: 0.52 in PCa vs 0.94 in BPH vs 2.71 in HC)

### Receiver operating characteristics analysis

**Fig 3:**
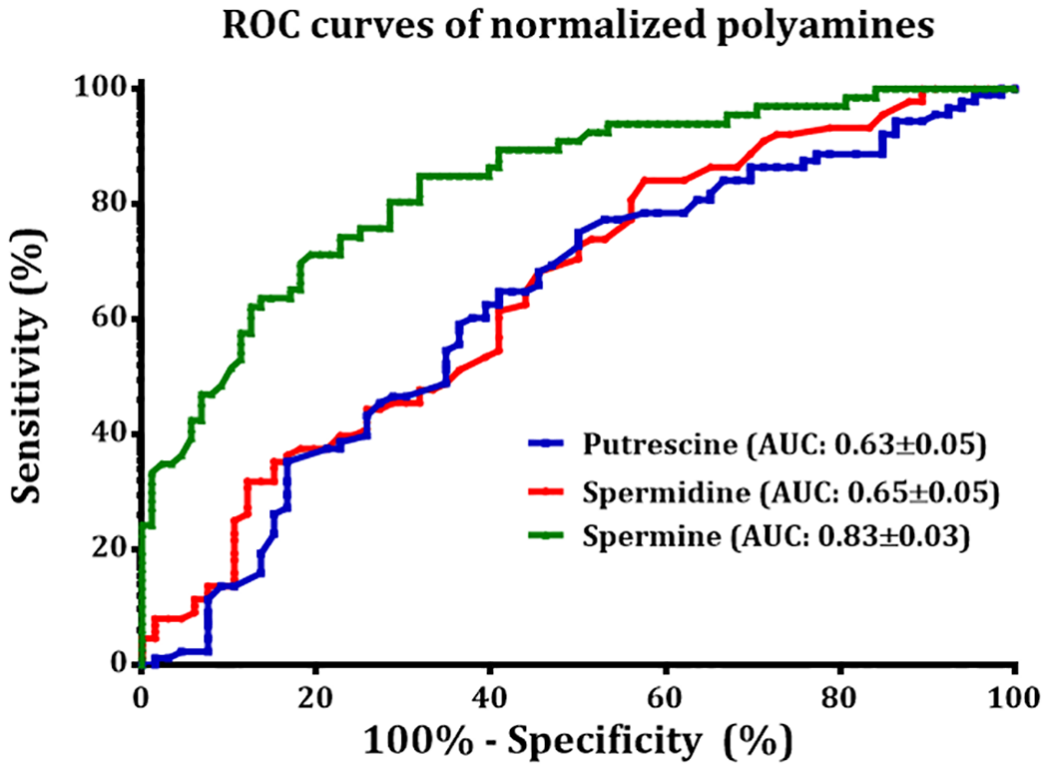
Receiver operating characteristic analysis for normalized Put, Spd and Spm values.

Fig 3 shows the ROC curves of the three normalized polyamines for evaluating the diagnostic power of the shortlisted polyamines for PCa diagnosis. The AUC for normalized Put, Spd and Spm were found to be 0.63±0.05, 0.65±0.05 and 0.83±0.03 respectively. The threshold values for Spm with the corresponding sensitivity and specificity are listed in S3 Table.

### Correlations studies

Correlations between urinary Spm had also been performed with patients’ clinicopathologic characteristics, like age, serum PSA, creatinine content, and prostate volume. However, all of them showed weak correlation with correlation coefficients < 0.1. (Data not shown)

## Discussion/Conclusions

The relationship between polyamines and cancer has long been investigated by scientists. It is generally believed that increase of polyamine levels in blood or urine reflect the enhanced levels of polyamine synthesis in rapid-growing cancer tissues/cells, since they are associated with increased cell proliferation, decreased apoptosis and increased expression of genes affecting tumor invasion and metastasis. [20, 25, 26, 27] Russell firstly reported the increase of urinary polyamines levels in various solid tumors, including ovarian teratoma, rectal carcinoma, lymphosarcoma, osteogenic sarcoma and acute myelocytic leukaemia. [16]. Kyoko Hiramatsu *et al*. reported an increase in *N*^*1*^, *N*^*12*^–Diacetylspermine in patients with early and late stage colorectal and breast cancers and established its role to be a novel marker for these cancers. [28] In cases of cervical cancer, Lee *et al*. had shown a significant elevation in polyamines level in Put, Spd and Spm. [17] For hepatic cancer, Liu *et al*. had monitored the level differences between polyamines, polyamine precursors and catabolites in both patients’ plasma and urines. [29] By analyzing these results carefully, different kinds of polyamines, in fact, showed different variations depending on the type of cancers. The claim of urinary polyamine levels elevating in cancer cases is not specific enough.

Nevertheless, very few reports focused on detecting the effects of PCa on urinary polyamines levels, which in turn might provide a potential diagnostic tool for this increasingly common cancer. In 1975 Fair *et al*. had reported a significant elevation of urinary Spd content in PCa patients by electrophoresis, but not Put and Spm. [30] Horn *et al*. analyzed urinary Spd and Put contents in patients with tumors in either breast, stomach, prostate, female genital tract, or metastatic carcinomas of unknown origins by LC with fluorometric detector in 1984 yielding an indeterminant conclusion. [31] With the advance of analytical field demonstrated in the current pilot study we evaluated the potential abilities of three natural polyamines: Put, Spd and Spm, as urinary biomarkers for screening of PCa by the powerful UPLC-MS/MS. Through a well validated method using separate deuterated internal standards for correcting matrix effects for each polyamine, we believe the analytical performance was much more reliable. Our results demonstrated that among the three polyamines, Spm was the best candidate to be employed as such a biomarker. A significant decrease of urinary Spm level had been observed which confers Spm specificity towards PCa monitoring. For Put and Spd, hardly could we find any significant changes between two subsets.

The observation of such a declined level in urinary Spm actually was reasonable from results of previous literature about PCa studies. Although only a limited number of tissue specimens had been examined, van der Graaf *et al*. reported a reduced Spm content in tumor prostatic tissues compared to normal and benign hyperplastic prostatic tissues by high performance liquid chromatography with fluorometric detector. [21] Swanson *et al*. also reported a decreased Spm level in prostate tissue samples by Proton high-resolution magic angle spinning nuclear magnetic resonance spectroscopy and quantitative histopathology. [32] High grade cancer prostate tissue could be distinguished from low grade cancer tissue by decreased concentrations of Spm and citrate, as reported by GF Giskeødegård *et al*. [33] Apart from direct monitoring of prostate tissue, Serkova *et al*. reported that in human expressed prostatic secretions, citrate, myo-inositol and Spm are potentially important markers of PCa, and all of them showed a decreased level in PCa patients compared to control samples. The AUC value reported in their study for Spm is 0.87 in EPS, which is close to the value discovered in this research, despite we only investigated patients with serum PSA value over 4.0 ng/ml. [34] With respect to these previous researches, a decrease in urinary Spm content could be foreseen because urine represents a fluid closely related to exfoliated cancer cells and secreted prostatic products from the prostate. [13] In essence, urine has the advantages of ready availability and non-invasive characters with which the prostate tissue samples and EPS cannot compare with. Therefore the discovery of a useful urine PCa biomarker is inspiring to the current medical situation for reducing unnecessary biopsies and arranging patients for appropriate therapies.

**Fig 4:**
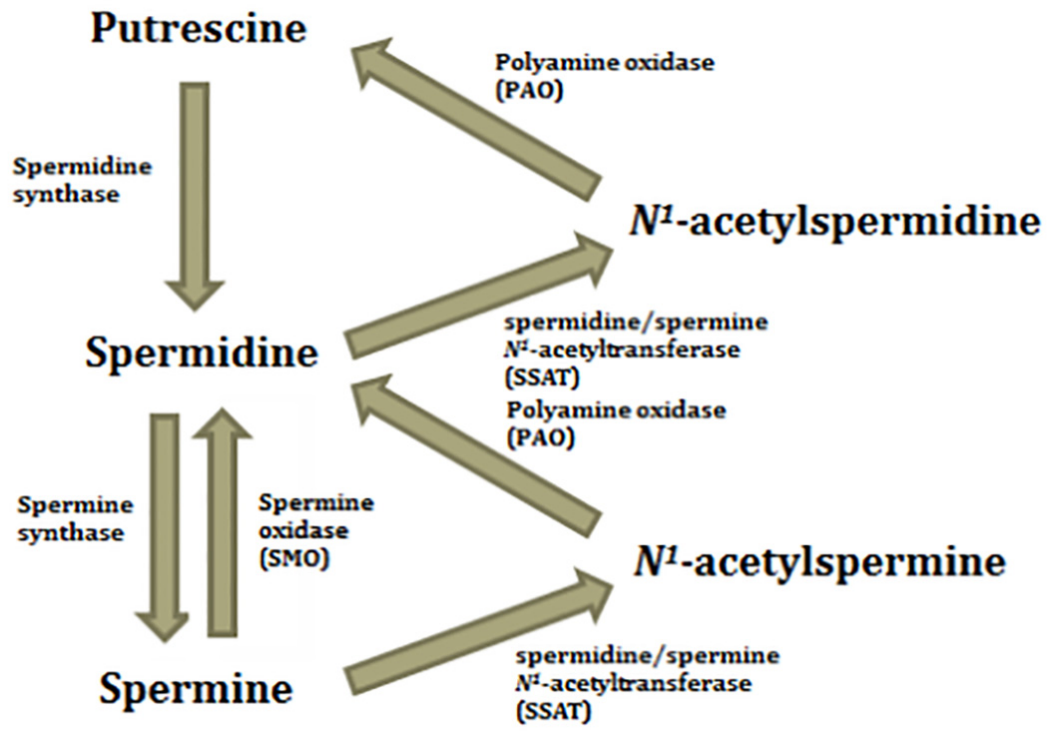
Polyamine metabolic pathway (focusing on Put, Spd and Spm only).

To explain the declined level of Spm in PCa patients, the exact mechanism lacks clear evidence and is still under research. Schipper *et al*. suggested a possible explanation that changes of cell organization caused by cancer cell proliferation finally result in a decreased luminal volume, which in turn reduces the amount of secreted compounds in prostate tissue, prostatic fluid or even urine. [20] But this could hardly explain why only urinary Spm level declined. Leo *et al*. reported that Spm was a proposed endogeneous inhibitor to prostate cancer growth, and a linear correlation was found between Spm content and the volume percentage of normal prostatic epithelial cells as quantified by histopathology. [35] And in recent studies it was suggested that dysregulation of polyamine metabolism, or more specifically polyamine catabolism, may be involved in carcinogenesis. Increases in spermine oxidase (SMO) and spermidine/spermine *N*^*1*^-acetyltransferase (SSAT) expression were observed in both precursor prostatic inflammatory atrophy lesions and early prostatic intraepithelial neoplastic lesions, which resulted in a depletion of Spm content. (See Fig 4) [36–38] This hypothesis is also supported by the observation of a significant increase in urinary diacetylspermine content in patients with urogenital malignancies resulted from the enzymatic action of SSAT, as reported by Hiramatsu, *et al*. [39] Therefore our observation of a decrease in urinary Spm is in line with previous findings and suggested mechanisms. For Spd, we hypothesized the action of SMO and SSAT counteracted each other so no significant changes were found.

Nevertheless, unlike what GF Giskeødegård *et al*. reported that prostatic Spm content can act as a biomarker to assess PCa aggressiveness [33], we could not give a determinant conclusion on whether urinary Spm shows similar cancer grade-differentiating ability. From our results, a drop in high grade cancer (GS = 8–10) was observed compared to low grade cancer (GS≤6), albeit not that significant. (1.23 in High grade vs 1.47 in low grade; *p* = 0.611) Instead, it acts like a diagnostic biomarker working in accordance with TRUSPB for PCa diagnosis.

Compare to the currently adopted serum PSA test, it is generally accepted that it leaves much to be desired as a primary screening test. It had been shown to cause over-diagnosis especially to patients showing value in the grey zone. [40] For example, serum PSA alone demonstrated fair sensitivity and specificity of 65% and 47% respectively. [41] Li *et al*. reported an even poorer sensitivity and specificity for it in their study (Sensitivity = 54.8%, Specificity = 57.1%, AUC = 0.684). [42] Another large-scale study by Ferro *et al*. showed that total PSA only gave AUC value of 0.52±0.07. [43] Our data, which focused on patients with PSA>4.0ng/ml, shows the best screening performance (AUC = 0.73±0.04; See S2 Fig), but it is still poorer than that of urinary Spm. Sensitivity and specificity were 67.05% and 68.75% respectively. Therefore urinary Spm is able to act as a secondary screening test to men with serum PSA>4.0ng/ml to differentiate PCa and non-cancerous cases including BPH for supplementing PSA test.

The current research is not without limitations. Firstly, all patients sample are obtained from one hospital in Hong Kong, while collecting samples from multi-center and multi-country may strengthen the outcome of our study. Secondly, diet control had not been performed to completely exclude the effect from food habit, despite Vargas et al. reported before no significant association was found between dietary polyamine intake and urinary polyamines measured. [44] Finally, as a pilot study, the number of samples being analyzed was still limited. Large scale study is needed to further confirm the observation and mechanism in the future.

To conclude on the basis of this pilot study, the potential roles of the three main urinary polyamines as PCa biomarkers were evaluated. Among Put, Spd and Spm, Spm demonstrated an outstanding diagnostic performance for PCa, in particular for patients with elevated serum PSA level, upon comparison of their levels in PCa and BPH patients. Its AUC value is 0.83±0.03. This work takes a step towards tackling the current medical challenge of poor specificity of the serum PSA test.

## Supporting Information

S1 Fig Calibration graphs of polyamines. (A) Put (r^2^ = 0.9996) (B) Spd (r^2^ = 0.9993) (C) Spm (r^2^ = 0.9995).
(TIF)

S2 Fig Receiver operating characteristics curve for serum PSA test. (TIF)

S1 Table MRM transitions, dwell time, fragmentor, collision energy and cell acceletor voltage for Put, Spd, Spm and their corresponding internal standards. (* Denoted the quantifier transitions)
(DOC)

S2 Table Summary of creatinine results from all patients. (DOC)

S3 Table Sensitivity and Specificity for normalized Spm at different threshold values. (DOC)
