# Supplementary figures and images for: Urinary Polyamines: A Pilot Study on Their Roles as Prostate Cancer Detection Biomarkers
Source: PLoS One. 2016 Sep 6;11(9):e0162217. doi: 10.1371/journal.pone.0162217 (PMC5012650; doi:10.1371/journal.pone.0162217)

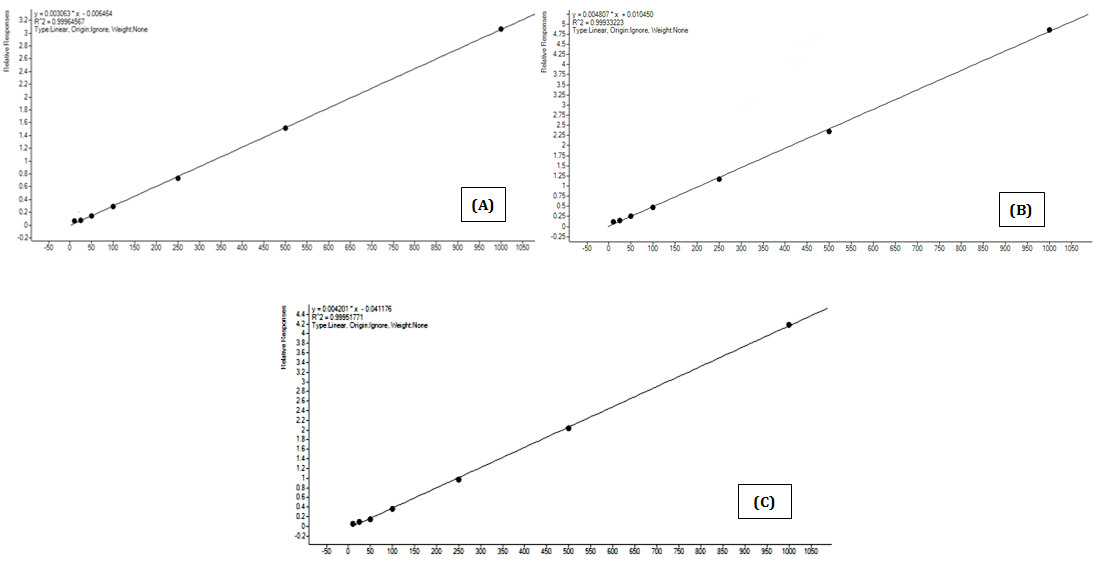

Supplement: S1 Fig — (A) Put (r2 = 0.9996) (B) Spd (r2 = 0.9993) (C) Spm (r2 = 0.9995). (TIF) [file pone.0162217.s001.tif]

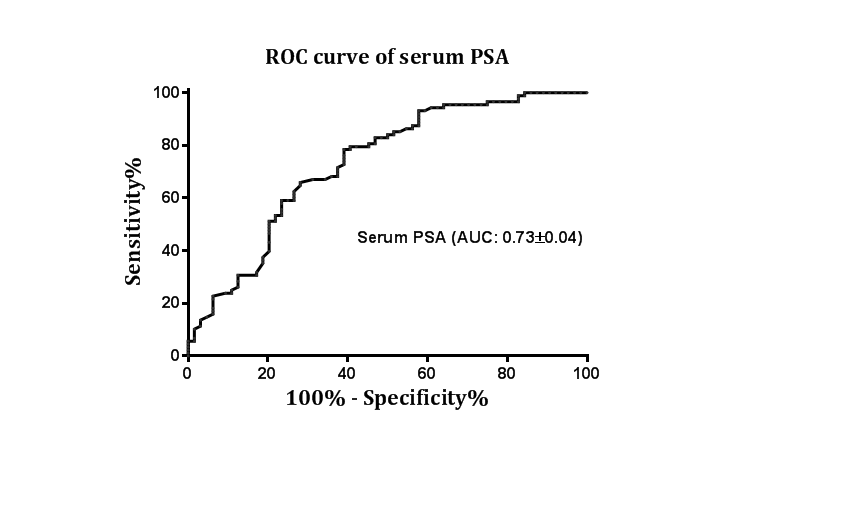

Supplement: S2 Fig — (TIF) [file pone.0162217.s002.tif]
