# Supplementary material for: Urinary Polyamines: A Pilot Study on Their Roles as Prostate Cancer Detection Biomarkers
Source: PLoS One. 2016 Sep 6;11(9):e0162217. doi: 10.1371/journal.pone.0162217 (PMC5012650; doi:10.1371/journal.pone.0162217)
Supplement: S1 Table — (* Denoted the quantifier transitions) (DOC) [file pone.0162217.s003.doc]

**Urinary Polyamines: A Pilot Study on Their Roles as Prostate Cancer Detection Biomarkers –**

**Tables S1**

Tik-Hung Tsoi^1^, Chi-Fai Chan^1^, Wai-Lun Chan^2^, Ka-Fung Chiu^3^, Wing-Tak Wong^1^*, Chi-Fai Ng^3^*, Ka-Leung Wong^4^*

^1^Department of Applied Biology and Chemical Technology, The Hong Kong Polytechnic University, Hung Hom, Hong Kong SAR.

^2^Department of Chemistry, National University of Singapore, 21 Lower Kent Ridge Road, Singapore.

^3^SH Ho Urology Centre, Division of Urology, Department of Surgery, The Chinese University of Hong Kong, Shatin, N.T., Hong Kong SAR

^4^Department of Chemistry, Hong Kong Baptist University, Kowloon Tong, Hong Kong SAR.

**Table S1. MRM transitions, dwell time, fragmentor, collision energy and cell accelerator voltage for Put, Spd, Spm and their corresponding internal standards**

|  | **Precusor ion** | **Daughter ion** | **Dwell time (ms)** | **Fragmentor (V)** | **Collision energy (V)** | **Cell Accelerator Voltage (V)** |
| --- | --- | --- | --- | --- | --- | --- |
|  |
| **Putrescine** | 89 | 72* | 100 | 70 | 6 | 3 |
| **Putrescine - d_8_** | 97 | 80* | 100 | 70 | 6 | 3 |
|  |  |  |  |  |  |  |
| **Spermidine** | 146 | 112 | 100 | 90 | 10 | 3 |
|  | 146 | 72* | 100 | 90 | 15 | 3 |
| **Spermidine - d_8_** | 154 | 120 | 100 | 90 | 10 | 3 |
|  | 154 | 80* | 100 | 90 | 15 | 3 |
|  |  |  |  |  |  |  |
| **Spermine** | 203 | 129 | 100 | 90 | 10 | 3 |
|  | 203 | 112* | 100 | 90 | 15 | 3 |
| **Spermine - d_8_** | 211 | 137 | 100 | 90 | 10 | 3 |
|  | 211 | 120* | 100 | 90 | 15 | 3 |

* Denoted the quantifier transitions
