# Supplementary material for: Urinary Polyamines: A Pilot Study on Their Roles as Prostate Cancer Detection Biomarkers
Source: PLoS One. 2016 Sep 6;11(9):e0162217. doi: 10.1371/journal.pone.0162217 (PMC5012650; doi:10.1371/journal.pone.0162217)
Supplement: S2 Table — (DOC) [file pone.0162217.s004.doc]

**Urinary Polyamines: A Pilot Study on Their Roles as Prostate Cancer Detection Biomarkers –**

**Tables S2**

Tik-Hung Tsoi^1^, Chi-Fai Chan^1^, Wai-Lun Chan^2^, Ka-Fung Chiu^3^, Wing-Tak Wong^1^*, Chi-Fai Ng^3^*, Ka-Leung Wong^4^*

^1^Department of Applied Biology and Chemical Technology, The Hong Kong Polytechnic University, Hung Hom, Hong Kong SAR.

^2^Department of Chemistry, National University of Singapore, 21 Lower Kent Ridge Road, Singapore.

^3^SH Ho Urology Centre, Division of Urology, Department of Surgery, The Chinese University of Hong Kong, Shatin, N.T., Hong Kong SAR

^4^Department of Chemistry, Hong Kong Baptist University, Kowloon Tong, Hong Kong SAR.

**Table S2. Summary of creatinine results from all patients**

|  | **PCa**  **(n=66)** | **BPH**  **(n=88)** | **HC**  **(n=11)** | ***p* value (PCa vs BPH)** | ***p***  **value (PCa vs**  **HC)** | ***p* value (BPH vs HC)** |
| --- | --- | --- | --- | --- | --- | --- |
| **Creatinine (ppm)** |  |  |  |  |  |  |
| **Mean(SEM)** | 828.30(76.82) | 970.40(69.06) | 1143(269.40) | 0.173 | 0.151 | 0.427 |
| **Median**  **Range** | 671.31  119.57-3102.61 | 751.80  171.43-3025.24 | 951.98  174.16-3183.77 |  |  |  |
