# Supplementary material for: Urinary Polyamines: A Pilot Study on Their Roles as Prostate Cancer Detection Biomarkers
Source: PLoS One. 2016 Sep 6;11(9):e0162217. doi: 10.1371/journal.pone.0162217 (PMC5012650; doi:10.1371/journal.pone.0162217)
Supplement: S3 Table — (DOC) [file pone.0162217.s005.doc]

**Urinary Polyamines: A Pilot Study on Their Roles as Prostate Cancer Detection Biomarkers –**

**Tables S3**

Tik-Hung Tsoi^1^, Chi-Fai Chan^1^, Wai-Lun Chan^2^, Ka-Fung Chiu^3^, Wing-Tak Wong^1^*, Chi-Fai Ng^3^*, Ka-Leung Wong^4^*

^1^Department of Applied Biology and Chemical Technology, The Hong Kong Polytechnic University, Hung Hom, Hong Kong SAR.

^2^Department of Chemistry, National University of Singapore, 21 Lower Kent Ridge Road, Singapore.

^3^SH Ho Urology Centre, Division of Urology, Department of Surgery, The Chinese University of Hong Kong, Shatin, N.T., Hong Kong SAR

^4^Department of Chemistry, Hong Kong Baptist University, Kowloon Tong, Hong Kong SAR.

**Table S3. Sensitivity and Specificity for normalized Spm at different threshold values**

| **Threshold** | **Sensitivity (%)** | **95%CI** | **Specificity (%)** | **95%CI** |
| --- | --- | --- | --- | --- |
| < 0.0800   | < 0.1150 | | --- | | < 0.1350 | | < 0.1800 | | < 0.2400 | | < 0.2900 | | < 0.3150 | | < 0.3250 | | < 0.3350 | | < 0.3550 | | < 0.3750 | | < 0.4000 | | < 0.4300 | | < 0.4450 | | < 0.4600 | | < 0.4800 | | < 0.5000 | | < 0.5200 | | < 0.5350 | | < 0.5500 | | < 0.5700 | | < 0.6050 | | < 0.6350 | | < 0.6600 | | < 0.6850 | | < 0.6950 | | < 0.7200 | | < 0.7650 | | < 0.7950 | | < 0.8200 | | < 0.8450 | | < 0.8550 | | < 0.8650 | | < 0.8800 | | < 0.9100 | | < 0.9400 | | < 0.9650 | | < 0.9850 | | < 1.035 | | < 1.110 | | < 1.145 | | < 1.175 | | < 1.205 | | < 1.225 | | < 1.250 | | < 1.280 | | < 1.305 | | < 1.320 | | < 1.335 | | < 1.365 | | < 1.425 | | < 1.480 | | < 1.520 | | < 1.560 | | < 1.585 | | < 1.620 | | < 1.655 | | < 1.705 | | < 1.830 | | < 1.940 | | < 1.990 | | < 2.045 | | < 2.105 | | < 2.175 | | < 2.235 | | < 2.255 | | < 2.270 | | < 2.325 | | < 2.390 | | < 2.415 | | < 2.435 | | < 2.455 | | < 2.465 | | < 2.485 | | < 2.510 | | < 2.585 | | < 2.665 | | < 2.715 | | < 2.770 | | < 2.800 | | < 2.820 | | < 2.845 | | < 2.895 | | < 2.935 | | < 2.965 | | < 3.110 | | < 3.235 | | < 3.245 | | < 3.380 | | < 3.555 | | < 3.625 | | < 3.760 | | < 3.920 | | < 4.055 | | < 4.170 | | < 4.205 | | < 4.235 | | < 4.290 | | < 4.430 | | < 4.560 | | < 4.595 | | < 4.625 | | < 4.695 | | < 4.880 | | < 5.180 | | < 5.440 | | < 5.700 | | < 6.055 | | < 6.305 | | < 6.435 | | < 6.555 | | < 6.685 | | < 7.000 | | < 7.270 | | < 7.310 | | < 7.540 | | < 7.885 | | < 8.060 | | < 8.130 | | < 8.245 | | < 8.495 | | < 8.755 | | < 8.880 | | < 9.040 | | < 9.335 | | < 9.520 | | < 9.560 | | < 9.700 | | < 10.60 | | < 11.85 | | < 12.38 | | < 12.63 | | < 12.93 | | < 13.15 | | < 13.29 | | < 16.50 | | < 19.83 | | < 20.11 | | < 22.08 | | < 25.01 | | < 28.75 | | < 31.57 | | 1.515   | 3.03 | | --- | | 4.545 | | 6.061 | | 7.576 | | 9.091 | | 10.61 | | 12.12 | | 13.64 | | 15.15 | | 16.67 | | 18.18 | | 19.7 | | 22.73 | | 24.24 | | 24.24 | | 27.27 | | 28.79 | | 30.3 | | 31.82 | | 33.33 | | 34.85 | | 34.85 | | 36.36 | | 39.39 | | 40.91 | | 42.42 | | 42.42 | | 45.45 | | 46.97 | | 46.97 | | 48.48 | | 51.52 | | 53.03 | | 54.55 | | 56.06 | | 57.58 | | 57.58 | | 60.61 | | 62.12 | | 62.12 | | 63.64 | | 63.64 | | 63.64 | | 65.15 | | 65.15 | | 65.15 | | 66.67 | | 68.18 | | 69.7 | | 71.21 | | 71.21 | | 71.21 | | 71.21 | | 72.73 | | 72.73 | | 74.24 | | 74.24 | | 75.76 | | 75.76 | | 75.76 | | 77.27 | | 80.3 | | 80.3 | | 80.3 | | 80.3 | | 81.82 | | 84.85 | | 84.85 | | 84.85 | | 84.85 | | 84.85 | | 84.85 | | 84.85 | | 84.85 | | 86.36 | | 86.36 | | 87.88 | | 89.39 | | 89.39 | | 89.39 | | 89.39 | | 89.39 | | 89.39 | | 89.39 | | 90.91 | | 90.91 | | 90.91 | | 92.42 | | 92.42 | | 92.42 | | 93.94 | | 93.94 | | 93.94 | | 93.94 | | 93.94 | | 93.94 | | 93.94 | | 93.94 | | 93.94 | | 93.94 | | 93.94 | | 93.94 | | 93.94 | | 93.94 | | 95.45 | | 95.45 | | 95.45 | | 95.45 | | 95.45 | | 95.45 | | 96.97 | | 96.97 | | 96.97 | | 96.97 | | 96.97 | | 96.97 | | 96.97 | | 96.97 | | 96.97 | | 96.97 | | 98.48 | | 98.48 | | 98.48 | | 98.48 | | 98.48 | | 98.48 | | 100 | | 100 | | 100 | | 100 | | 100 | | 100 | | 100 | | 100 | | 100 | | 100 | | 100 | | 100 | | 100 | | 100 | | 100 | | 0.03835% to 8.155%   | 0.3691% to 10.52% | | --- | | 0.9474% to 12.71% | | 1.676% to 14.80% | | 2.506% to 16.80% | | 3.410% to 18.74% | | 4.372% to 20.64% | | 5.381% to 22.49% | | 6.430% to 24.31% | | 7.512% to 26.10% | | 8.625% to 27.87% | | 9.763% to 29.61% | | 10.93% to 31.32% | | 13.31% to 34.70% | | 14.54% to 36.36% | | 14.54% to 36.36% | | 17.03% to 39.64% | | 18.30% to 41.25% | | 19.59% to 42.85% | | 20.89% to 44.44% | | 22.20% to 46.01% | | 23.53% to 47.58% | | 23.53% to 47.58% | | 24.87% to 49.13% | | 27.58% to 52.19% | | 28.95% to 53.71% | | 30.34% to 55.21% | | 30.34% to 55.21% | | 33.14% to 58.19% | | 34.56% to 59.66% | | 34.56% to 59.66% | | 35.99% to 61.12% | | 38.88% to 64.01% | | 40.34% to 65.44% | | 41.81% to 66.86% | | 43.30% to 68.26% | | 44.79% to 69.66% | | 44.79% to 69.66% | | 47.81% to 72.42% | | 49.34% to 73.78% | | 49.34% to 73.78% | | 50.87% to 75.13% | | 50.87% to 75.13% | | 50.87% to 75.13% | | 52.42% to 76.47% | | 52.42% to 76.47% | | 52.42% to 76.47% | | 53.99% to 77.80% | | 55.56% to 79.11% | | 57.15% to 80.41% | | 58.75% to 81.70% | | 58.75% to 81.70% | | 58.75% to 81.70% | | 58.75% to 81.70% | | 60.36% to 82.97% | | 60.36% to 82.97% | | 61.99% to 84.22% | | 61.99% to 84.22% | | 63.64% to 85.46% | | 63.64% to 85.46% | | 63.64% to 85.46% | | 65.30% to 86.69% | | 68.68% to 89.07% | | 68.68% to 89.07% | | 68.68% to 89.07% | | 68.68% to 89.07% | | 70.39% to 90.24% | | 73.90% to 92.49% | | 73.90% to 92.49% | | 73.90% to 92.49% | | 73.90% to 92.49% | | 73.90% to 92.49% | | 73.90% to 92.49% | | 73.90% to 92.49% | | 73.90% to 92.49% | | 75.69% to 93.57% | | 75.69% to 93.57% | | 77.51% to 94.62% | | 79.36% to 95.63% | | 79.36% to 95.63% | | 79.36% to 95.63% | | 79.36% to 95.63% | | 79.36% to 95.63% | | 79.36% to 95.63% | | 79.36% to 95.63% | | 81.26% to 96.59% | | 81.26% to 96.59% | | 81.26% to 96.59% | | 83.20% to 97.49% | | 83.20% to 97.49% | | 83.20% to 97.49% | | 85.20% to 98.32% | | 85.20% to 98.32% | | 85.20% to 98.32% | | 85.20% to 98.32% | | 85.20% to 98.32% | | 85.20% to 98.32% | | 85.20% to 98.32% | | 85.20% to 98.32% | | 85.20% to 98.32% | | 85.20% to 98.32% | | 85.20% to 98.32% | | 85.20% to 98.32% | | 85.20% to 98.32% | | 85.20% to 98.32% | | 87.29% to 99.05% | | 87.29% to 99.05% | | 87.29% to 99.05% | | 87.29% to 99.05% | | 87.29% to 99.05% | | 87.29% to 99.05% | | 89.48% to 99.63% | | 89.48% to 99.63% | | 89.48% to 99.63% | | 89.48% to 99.63% | | 89.48% to 99.63% | | 89.48% to 99.63% | | 89.48% to 99.63% | | 89.48% to 99.63% | | 89.48% to 99.63% | | 89.48% to 99.63% | | 91.84% to 99.96% | | 91.84% to 99.96% | | 91.84% to 99.96% | | 91.84% to 99.96% | | 91.84% to 99.96% | | 91.84% to 99.96% | | 94.56% to 100.0% | | 94.56% to 100.0% | | 94.56% to 100.0% | | 94.56% to 100.0% | | 94.56% to 100.0% | | 94.56% to 100.0% | | 94.56% to 100.0% | | 94.56% to 100.0% | | 94.56% to 100.0% | | 94.56% to 100.0% | | 94.56% to 100.0% | | 94.56% to 100.0% | | 94.56% to 100.0% | | 94.56% to 100.0% | | 94.56% to 100.0% | | 100.   | 100 | | --- | | 100 | | 100 | | 100 | | 100 | | 100 | | 100 | | 100 | | 100 | | 100 | | 100 | | 100 | | 100 | | 100 | | 98.99 | | 98.99 | | 98.99 | | 98.99 | | 98.99 | | 98.99 | | 97.98 | | 95.96 | | 94.95 | | 93.94 | | 93.94 | | 93.94 | | 92.93 | | 92.93 | | 92.93 | | 91.92 | | 90.91 | | 89.9 | | 88.89 | | 88.89 | | 88.89 | | 88.89 | | 87.88 | | 86.87 | | 86.87 | | 85.86 | | 85.86 | | 84.85 | | 82.83 | | 82.83 | | 81.82 | | 80.81 | | 80.81 | | 80.81 | | 80.81 | | 79.8 | | 78.79 | | 77.78 | | 76.77 | | 76.77 | | 75.76 | | 75.76 | | 73.74 | | 73.74 | | 71.72 | | 70.71 | | 70.71 | | 70.71 | | 69.7 | | 68.69 | | 67.68 | | 67.68 | | 67.68 | | 66.67 | | 65.66 | | 64.65 | | 63.64 | | 62.63 | | 61.62 | | 60.61 | | 60.61 | | 59.6 | | 59.6 | | 59.6 | | 58.59 | | 57.58 | | 56.57 | | 55.56 | | 54.55 | | 53.54 | | 53.54 | | 52.53 | | 51.52 | | 50.51 | | 49.49 | | 48.48 | | 48.48 | | 47.47 | | 46.46 | | 45.45 | | 44.44 | | 43.43 | | 42.42 | | 41.41 | | 40.4 | | 39.39 | | 38.38 | | 37.37 | | 36.36 | | 35.35 | | 35.35 | | 34.34 | | 33.33 | | 32.32 | | 31.31 | | 30.3 | | 30.3 | | 29.29 | | 28.28 | | 27.27 | | 26.26 | | 25.25 | | 24.24 | | 23.23 | | 21.21 | | 20.2 | | 20.2 | | 19.19 | | 18.18 | | 17.17 | | 16.16 | | 15.15 | | 15.15 | | 14.14 | | 13.13 | | 12.12 | | 11.11 | | 10.1 | | 9.091 | | 8.081 | | 7.071 | | 6.061 | | 5.051 | | 4.04 | | 3.03 | | 2.02 | | 1.01 | | 96.34% to 100.0%   | 96.34% to 100.0% | | --- | | 96.34% to 100.0% | | 96.34% to 100.0% | | 96.34% to 100.0% | | 96.34% to 100.0% | | 96.34% to 100.0% | | 96.34% to 100.0% | | 96.34% to 100.0% | | 96.34% to 100.0% | | 96.34% to 100.0% | | 96.34% to 100.0% | | 96.34% to 100.0% | | 96.34% to 100.0% | | 96.34% to 100.0% | | 94.50% to 99.97% | | 94.50% to 99.97% | | 94.50% to 99.97% | | 94.50% to 99.97% | | 94.50% to 99.97% | | 94.50% to 99.97% | | 92.89% to 99.75% | | 89.98% to 98.89% | | 88.61% to 98.34% | | 87.27% to 97.74% | | 87.27% to 97.74% | | 87.27% to 97.74% | | 85.97% to 97.11% | | 85.97% to 97.11% | | 85.97% to 97.11% | | 84.70% to 96.45% | | 83.44% to 95.76% | | 82.21% to 95.05% | | 80.99% to 94.32% | | 80.99% to 94.32% | | 80.99% to 94.32% | | 80.99% to 94.32% | | 79.78% to 93.58% | | 78.59% to 92.82% | | 78.59% to 92.82% | | 77.41% to 92.05% | | 77.41% to 92.05% | | 76.24% to 91.26% | | 73.94% to 89.67% | | 73.94% to 89.67% | | 72.80% to 88.85% | | 71.66% to 88.03% | | 71.66% to 88.03% | | 71.66% to 88.03% | | 71.66% to 88.03% | | 70.54% to 87.20% | | 69.42% to 86.36% | | 68.31% to 85.52% | | 67.21% to 84.67% | | 67.21% to 84.67% | | 66.11% to 83.81% | | 66.11% to 83.81% | | 63.93% to 82.07% | | 63.93% to 82.07% | | 61.78% to 80.31% | | 60.71% to 79.43% | | 60.71% to 79.43% | | 60.71% to 79.43% | | 59.64% to 78.53% | | 58.59% to 77.64% | | 57.53% to 76.73% | | 57.53% to 76.73% | | 57.53% to 76.73% | | 56.48% to 75.82% | | 55.44% to 74.91% | | 54.40% to 73.99% | | 53.36% to 73.07% | | 52.33% to 72.15% | | 51.30% to 71.22% | | 50.28% to 70.28% | | 50.28% to 70.28% | | 49.26% to 69.34% | | 49.26% to 69.34% | | 49.26% to 69.34% | | 48.24% to 68.40% | | 47.23% to 67.45% | | 46.23% to 66.50% | | 45.22% to 65.55% | | 44.22% to 64.59% | | 43.23% to 63.62% | | 43.23% to 63.62% | | 42.24% to 62.66% | | 41.25% to 61.68% | | 40.27% to 60.71% | | 39.29% to 59.73% | | 38.32% to 58.75% | | 38.32% to 58.75% | | 37.34% to 57.76% | | 36.38% to 56.77% | | 35.41% to 55.78% | | 34.45% to 54.78% | | 33.50% to 53.77% | | 32.55% to 52.77% | | 31.60% to 51.76% | | 30.66% to 50.74% | | 29.72% to 49.72% | | 28.78% to 48.70% | | 27.85% to 47.67% | | 26.93% to 46.64% | | 26.01% to 45.60% | | 26.01% to 45.60% | | 25.09% to 44.56% | | 24.18% to 43.52% | | 23.27% to 42.47% | | 22.36% to 41.41% | | 21.47% to 40.36% | | 21.47% to 40.36% | | 20.57% to 39.29% | | 19.69% to 38.22% | | 18.80% to 37.15% | | 17.93% to 36.07% | | 17.06% to 34.98% | | 16.19% to 33.89% | | 15.33% to 32.79% | | 13.64% to 30.58% | | 12.80% to 29.46% | | 12.80% to 29.46% | | 11.97% to 28.34% | | 11.15% to 27.20% | | 10.33% to 26.06% | | 9.530% to 24.91% | | 8.736% to 23.76% | | 8.736% to 23.76% | | 7.953% to 22.59% | | 7.181% to 21.41% | | 6.423% to 20.22% | | 5.679% to 19.01% | | 4.951% to 17.79% | | 4.242% to 16.56% | | 3.553% to 15.30% | | 2.890% to 14.03% | | 2.256% to 12.73% | | 1.660% to 11.39% | | 1.112% to 10.02% | | 0.6293% to 8.601% | | 0.2456% to 7.108% | | 0.02557% to5.500% | |
